# Annotations

**Published:** 1903-07-04

**Authors:** 


### Annotations.

### The Power of the Penny.

A story connected with the German colony for epileptics, at Bielefeld, illustrates in a remarkable manner the power for good that dwells in little things when a generous motive lies behind them. One day some years ago, and after the service in the church belonging to the colony had ended, a man sought out the pastor, and said to him:—"I have come here to-day to pay a debt. Two years ago I was at this church, and during the service several of the children had fits, and had to be taken out and attended to. This made me think of the four healthy children whom God has given me, and I resolved that while they were in health I would give for each one of them a groschen (a little more than a penny) a year to this colony. I was not here last year, so now I want to pay four groschen for last year, four for this, and, lest I should not be able to come again soon, I will give you now the four groschen for the next two years." So saying, he laid before the pastor 16 groschen. It was the free-will offering of a poor man, and therefore, we may be sure, accepted in the spirit in which it was given by the Great Physician. But there is an earthly sequel. The pastor told the touching little story, and by degrees it got published about all Germany. It aroused a response in the heart of many parents, and groschen poured in from far and near. A regular "Groschen Fund" was established, and out of the proceeds of this there was presently built at Bielefeld a new hospital for epileptic children. It would be interesting to know how much a "Penny Fund" for every healthy child would bring from the parents of England to-day. At least the story shows how much may be done by all, even the poorest, giving what they can spare for the help of the sick and suffering.

### Panaceas for Youthful Crime.

In the annals of recent crime there has been nothing more pitiful than the trial of an eight-year-old boy for murder. But it is curious to note that, as from one text many sermons can be preached, several people have different nostrums to offer wherewith to prevent the repetition of crimes such as that of which Patrick Knowles is accused. The Dowager Countess of Portsmouth asks for the introduction into Parliament of a Bill forbidding children to enter slaughter-houses. This is already forbidden in London, and it is certain that when children do visit such places it is without the knowledge, not to say approval, of their parents. But a normal child may do these things without becoming a murderer, just as a normal visitor to Chicago may go to see the pigs killed in Armour's stock-yards without similar result. The criminal is a criminal because he is not normal, and in the records of youthful crime there are many instances of murders done by children who had no preliminary training in the ways of bloodshed. The *Spectator* also has its panacea—the teaching of physical drill. Abundance of physical exercise is supposed to turn to wholesome account the energy which otherwise might come out in morbid forms. In face of recent "ragging" exposures, we may reasonably doubt if physical efficiency has always the wholesome and refining influence with which it is credited, and it is possible that our educationists mistake the place which exercise has in the making of a man. Whether drill and physical exercises are equally good for children of the class of Knowles, who are not well-fed and healthy, is not so certain. The figures quoted by Sir John Gorst regarding the children of Edinburgh seem to show that for a great proportion of these drill is not only useless but injurious, unless physical education goes hand in hand with pure air and sufficient food. A hungry child may be worse-tempered and more savage after his hour of drill than before, if the means of satisfying his hunger are not within his reach. But if he is normal, the drill will not incite him to murder, nor will it keep him from it if he is abnormal.

### The Hospitals of Utopia.

As the Archbishop of Canterbury reminded us the other day, the provision of great hospitals for the sick in the capital city of Utopia is a memorable thing in Sir Thomas More's romance. They were to be hospitals both for infectious diseases and for ordinary sickness. It was an unaided flight of the imagination: for at that time, besides the old monastic charities of St. Bartholomew in the City and St. Thomas in the Borough, London had only a few poor shelters for "lepers," or privileged beggars by the wayside, on the several main roads approaching the City—at the bar or toll-gate of Southwark, at Hackney, at Highgate, at St. Giles' outside Holborn Bar, and at Knightsbridge. The two miserable pest-houses for the plague-stricken, in Finsbury and in Tothill Fields, Westminster, had not then come into existence. Sir Thomas conceived the hospitals of his model city on a great scale. They were not for the sick poor only, but for the sick citizens of every degree, excepting (we are to infer) princes, bishops, ambassadors, and "tanibores." There were to be four of them, one for each quarter of the city, a little without the walls, "so big, so wide, so ample, and so large that they may seem four little towns." They were so well appointed and provided, and so well served by the continual presence of cunning physicians, "that, though no man be sent thither against his will, yet notwithstanding there is no sick person in all the city that had not rather lie there than at home in his own house." Sir Thomas More being, for the nonce, an out-and-out socialist, is not at all troubled by the vision of the private medical practitioners crying out against the interference of charity with their legitimate business. Everything was done by the community for the community; and the cunning physicians who were continually present in these vast institutions were doubtless paid quarterly by warrants on the Treasury, which is a much more agreeable mode of remuneration than keeping accounts and sending in bills at Christmas. Nearly two centuries passed before London made an attempt at collective treatment of the sick, in the form of the very modest Dispensary (made famous by Garth) located in a single room in Warwick Lane. But even that humble enterprise was wrecked upon the rock which the great socialist vision ignored—namely, the interests of the apothecaries, as the general practitioners were then called.

